# Call for papers for the Special Issue: “British and Eire Biophysics”

**DOI:** 10.1007/s12551-024-01257-8

**Published:** 2024-12-14

**Authors:** Stephen Harding, Anthony Watts

**Affiliations:** 1 University of Nottingham, Nottingham, UK; 2 University of Oxford, Oxford, UK

**Keywords:** British & Eire Biophysics Special

## Abstract

This is a call for papers for a special issue of Biophysical Reviews devoted to British and Eire Biophysics, and follows a successful special issue dedicated to Japanese Biophysics published in 2020. Our objective is to have under one volume a selection of articles that can give the wider scientific community a firm insight into current developments in biophysical science in Britain and Eire.

The Special Issue will combine a number of short commentary articles from prominent members of the British Biophysical Society (BBS) together with a number of short and longer review articles based (but not exclusively) on the recent meeting of the BBS in Swansea and other related meetings such as the Midlands Biophysical Symposium held at Leicester. In addition, all Honorary Fellows of the BBS will be invited to submit a review article of their choosing. All participants, and indeed all researchers who are working or have worked in laboratories in the UK and Eire, are cordially invited to contribute a review. Graduate students—particularly those who have recently (or are about to) complete their studies—are also encouraged to submit a review based on the introductory chapters of their PhD theses.

**Submission deadline**: April 15th, 2025. Those contributing are requested to accept this invitation by February 15th. When submitting your article, please choose the correct Special Issue assignment.

**Special Issue Editors**: Stephen Harding and Anthony Watts.

**Contact:** steve.harding@nottingham.ac.uk; anthony.watts@bioch.ox.ac.uk.

**Submission format**: Two types of review formats will be considered: a short review (3000–4000 words, ~3 figures) or a long review (~ 10,000 words, ~ 10 figures). Also, Commentaries and Letters (200–600 words, 1 figure, ~ 10 references) are also requested from prominent members of the scientific community with these commentaries being of either an educational, historical or topical nature.

**About Biophysical Reviews**: “Biophysical Reviews” was launched in 2009 and is the official journal of the “International Union for Pure and Applied Biophysics”. *Biophysical Reviews* publishes both short and long review formats from key figures in the field. The majority of contributions are invited although interested authors are encouraged to discuss with the Editor the possibility of contributing a review article. *Biophysical Reviews* covers the entire field of biophysics, generally defined as the science of describing and defining biological phenomenon using the concepts and techniques of physics. This includes but is not limited by such areas as bioinformatics, biophysical methods and instrumentation, medical biophysics, biosystems, cell biophysics and cellular organization, macromolecule dynamics, structure and interactions, single molecule biophysics, membrane biophysics, channels and transportation.

## Journal homepage

http://www.springer.com/journal/12551.

## Online submission

https://submission.springernature.com/new-submission/12551/3

**Cost:** Biophysical Reviews is a hybrid open access journal and therefore operates on a two-track payment structure. The first track is completely free but requires that the article exist behind a paywall for approximately 6 months whilst the second track involves an open access charge of 2500 Euros. Both tracks give a highly desirable publishing outcome for the author. Please contact the Special Issue Editors for further description of this point.

